# Differences in Telemedicine Use for Patients With Diabetes in an Academic Versus Safety Net Health System: Retrospective Cohort Study

**DOI:** 10.2196/64635

**Published:** 2025-03-24

**Authors:** Jonathan J Shih, Magdalene Kuznia, Sarah Nouri, Elizabeth B Sherwin, Kathryn E Kemper, Anna D Rubinsky, Courtney R Lyles, Elaine C Khoong

**Affiliations:** 1 School of Medicine University of California, San Francisco San Francisco, CA United States; 2 School of Nursing University of California, San Francisco San Francisco, CA United States; 3 Division of Palliative Medicine Department of Medicine University of California, San Francisco San Francisco, CA United States; 4 Department of Epidemiology and Biostatistics University of California, San Francisco San Francisco, CA United States; 5 UCSF Action Research Center for Health Equity University of California, San Francisco San Francisco, CA United States; 6 Academic Research Services Information Technology University of California, San Francisco San Francisco, CA United States; 7 Center for Healthcare Policy and Research University of California, Davis Davis, CA United States; 8 Department of Public Heath Science University of California, Davis Davis, CA United States; 9 Department of Medicine Division of General Internal Medicine at Zuckerberg San Francisco General Hospital University of California, San Francisco San Francisco, CA United States

**Keywords:** telehealth, telemedicine, diabetes, delivery of health care, primary health care, health care utilization, health care disparities, chronic disease, COVID-19, safety net providers, public health emergency, older adults

## Abstract

**Background:**

The COVID-19 public health emergency catalyzed widespread adoption of both video- and audio-only telemedicine visits. This proliferation highlighted inequities in use by age, race and ethnicity, and preferred language. Few studies have investigated how differences in health system telemedicine implementation affected these inequities.

**Objective:**

This study aims to describe patients who used telemedicine during the public health emergency and identify predictors of telemedicine use across 2 health systems with different telemedicine implementations.

**Methods:**

This retrospective cohort study included adults with diabetes receiving primary care between July 2020 and March 2021 at 2 independent health systems in San Francisco, California. Participant sociodemographic characteristics, health information, and telemedicine utilization were acquired from electronic health records. The primary outcome was visit type (any audio or video telemedicine vs in-person only) during the study period. We used multivariable logistic regression to assess the association between visit type and key predictors associated with digital exclusion (age, race and ethnicity, preferred language, and neighborhood socioeconomic status), adjusting for baseline health. We included an interaction term to evaluate health system impact on each predictor and then stratified by health system (academic, which prioritized video-enabled visits, vs safety net, which prioritized audio-only visits).

**Results:**

Among 10,201 patients, we found higher odds of telemedicine use in the safety net system compared with the academic system (adjusted odds ratio [aOR] 2.94, 95% CI 2.48-3.48). Patients with younger age (18-34 years: aOR 2.55, 95% CI 1.63-3.97; 35-49 years: aOR 1.39, 95% CI 1.12-1.73 vs 75+ years) and Chinese-language preference (aOR 2.04, 95% CI 1.66-2.5 vs English) had higher odds of having a telemedicine visit. Non-Hispanic Asian (aOR 0.67, 95% CI 0.56-0.79), non-Hispanic Black (aOR 0.83, 95% CI 0.68-1), and Hispanic or Latine (aOR 0.76, 95% CI 0.61-0.95) patients had lower odds of having a telemedicine visit than non-Hispanic White patients. We found significant interactions between health system and age, race and ethnicity, and preferred language (*P*<.05). After stratifying by health system, several differences persisted in the academic system: non-Hispanic Asian (aOR 0.57, 95% CI 0.46-0.70) and Latine (aOR 0.67, 95% CI 0.50-0.91) patients had lower odds of a telemedicine visit, and younger age groups had higher odds (18-34 years: aOR 3.97, 95% CI 1.99-7.93; 35-49 years: aOR 1.86, 95% CI 1.36-2.56). In the safety net system, Chinese-speaking patients had higher odds of having a telemedicine visit (aOR 2.52, 95% CI 1.85-3.42).

**Conclusions:**

We found disparities in telemedicine utilization by age, race and ethnicity, and preferred language, primarily in the health system that used more video visits. While telemedicine expanded rapidly recently, certain populations remain at risk for digital exclusion. These findings suggest that system-level factors influence telemedicine adoption and implementation decisions impact accessibility for populations at risk for digital exclusion.

## Introduction

Prior to the COVID-19 public health emergency (PHE), the adoption of telemedicine (defined here as real-time virtual visits between patients and clinicians) in the United States was limited due to both policy and implementation challenges [1,2]. The PHE served as a catalyst for widespread adoption: telemedicine policies expanded coverage to include reimbursement parity for both video- and audio-only visits, with notably high adoption of audio-only visits in safety net settings [3-5]. The proliferation of telemedicine, however, also introduced important concerns about equity [6]. Persistent inequities in utilization and access have been documented, with studies indicating that older adults, racial and ethnic minority groups, individuals with limited English proficiency, and those with lower income are less likely to adopt telemedicine, particularly video-enabled services [7-12]. These patterns of underutilization highlight the broader issues of digital exclusion, driven by challenges such as limited digital literacy and lack of access to technology and web-based service [6,12].

However, an understudied aspect in these studies is how health system factors and their implementation of telemedicine impact these disparities. Audio visits, provided more frequently by community health centers and safety net systems, are predominantly used by low-income, older, or racially and ethnically minoritized patients [3]. It remains unclear whether these patterns in telemedicine utilization are a result of patient characteristics or reflective of health systems being underresourced. Understanding the drivers of these patterns of telemedicine utilization is crucial, as it informs the development of interventions that address disparities and enhance equitable distribution of telehealth services.

To address this gap in the literature, we evaluated telemedicine use in 2 distinct health systems in San Francisco, California: University of California, San Francisco (UCSF), an academic medical system, and San Francisco Health Network (SFHN), a safety net system. Telemedicine implementation during the PHE differed across the systems. UCSF started implementing video visits and training prior to the PHE and encouraged video as the predominant modality during the PHE, resulting in 50% of all visits (including in-person and telemedicine) being video visits [13]. In contrast, SFHN encountered delays in video implementation and did not prioritize video visits, resulting in telemedicine visits being primarily delivered as audio-only encounters (>95%) [14]. In this study, we aimed to (1) describe characteristics of patients with and without a telemedicine visit during the PHE in each health system, and (2) evaluate associations between key sociodemographic factors—age, race and ethnicity, preferred language, and neighborhood socioeconomic status (nSES)—and telemedicine use, including health system interaction effects. We sought to identify whether disparities in telemedicine use were driven by patient characteristics and by variations in the implementation of telemedicine across systems.

## Methods

### Study Setting and Data Source

This retrospective cohort study included patients with diabetes receiving primary care between July 1, 2020, and March 31, 2021, at 1 of 2 independent health care systems in San Francisco: UCSF and SFHN. UCSF is an academic tertiary care medical care center with 4 primary care practices. SFHN is a safety net health system for San Francisco County with 14 primary care practices.

### Study Sample

The study cohort was identified using electronic health records (EHRs) and included adult patients (aged 18 years and older) with diabetes mellitus who had at least 1 primary care or endocrinology encounter between July 1, 2020, and March 31, 2021 (hereafter referred to as the study period). Participants were required to have a diagnosis of diabetes prior to the beginning of the study period. In addition, participants must have been empaneled in primary care as of April 1, 2019 (approximately 1 year before start of the PHE), defined as having assigned a primary care provider and at least 1 primary care encounter within the 3 years prior to April 1, 2019. Details on this cohort are provided in a prior publication [14].

### Ethical Considerations

Data were deidentified for analysis and reporting. This study was approved by the UCSF institutional review board (#20-31253) with waiver of informed consent.

### Variables

Participants’ sociodemographic characteristics, health information (eg, comorbidities, vitals, and laboratory results), and telemedicine utilization were derived from EHRs. The primary outcome was defined as whether participants had any telemedicine visit (audio- or video-only visit vs in-person visit only) with their diabetes care team (primary care or endocrinology) during the study period. Key predictor variables were as follows: age (18-34, 35-49, 50-64, 65-74, and ≥75 years); race and ethnicity (non-Hispanic Asian, non-Hispanic Black, Hispanic or Latine, non-Hispanic White, and non-Hispanic other race); preferred language (English, Spanish, Chinese, and other); and nSES quintile at the census tract level based on geocoded residential address [14]. nSES is a validated composite SES measure that incorporates 5-year census tract level data from the 2013-2017 American Community Survey on income, education, poverty, employment, occupation, housing, and rent values [15]. nSES quintiles are based on the distribution of nSES across US Census tracts from Bay Area counties, with the first quintile (Q1) reflecting lowest nSES and the fifth quintile (Q5) reflecting highest nSES.

Covariates included average baseline hemoglobin A_1c_ control, baseline blood pressure (BP) control (≤120/80, 121/81-140/90, and >140/90 mm Hg), patient sex (male or female), insurance type (Medicare, Medicaid, commercial, Healthy Workers [county administered], or uninsured), patient portal enrollment (yes or no, as a proxy for digital access), and Charlson Comorbidity Index (CCI) score category (0-2 or 3+). Hemoglobin A_1c_ control, BP control, and CCI were used to adjust for differences in utilization based on medical need.

### Statistical Analysis

We described patient-level differences in telemedicine use associated with each predictor, overall and by health care system, using chi-square tests for categorical predictors and 2-tailed *t* tests for continuous predictors. Our regression analyses included a multivariable logistic regression model using the combined health system data to determine the adjusted effects of key predictors on telemedicine utilization and a second model using an interaction term to estimate the effect of health system on each of our key predictors (age, race and ethnicity, preferred language, and nSES). We calculated predicted margins for the significant key predictors. Next, we stratified models by health system. Each logistic regression model controlled for our listed covariates. Missing data for hemoglobin A_1c_ values and BP measurements were imputed using multiple imputation by chained equations [16]. Results were presented as adjusted odds ratios (aORs) with 95% CIs. Two-tailed significance was determined with α value of .05. All analyses were performed using Stata 17 [17].

## Results

### Included Participants

The final study cohort included a total of 10,201 adult patients with diabetes across the 2 health systems (Table 1 and Multimedia Appendix 1). As shown in Multimedia Appendix 1, there was different racial and ethnic, linguistic, and nSES distribution in the 2 health systems. The academic health system had mostly non-Hispanic White and non-Hispanic Asian patients (929/3623, 26%, and 1430/3623, 39%, respectively), while the safety net system had more non-Hispanic Asian and Hispanic or Latine patients (2302/6578, 35%, and 2244/6578, 34%, respectively). Most of the patients in the academic health system had an English-language preference (2935/3623, 81%); however, less than half of the patients at the safety net system preferred the English language (2847/6578, 43%). Furthermore, the safety net system mostly took care of patients living in the lowest nSES quintile (2551/6578, 39%), whereas the academic health system had about 20% (Q1: 640/3623, 17.7%; Q2: 682/3623, 188.%; Q3: 716/3623, 198.%; Q4: 883/3623, 24.4%; and Q5: 702/3623, 19.4%) of patients in each nSES quintile.

Of the 10,201 patients in the cohort, 81% (n=8305) had at least 1 telemedicine visit during the study period. Patients in the safety net system were more likely to use telemedicine than patients in the academic health system (5655/6578, 86% vs 2650/3623, 73%; *P*<.001). Among the combined health systems, there were significant differences in telemedicine use by age (*P*<.001), language preference (*P*<.001), and nSES quintile (*P*<.001). Within the academic health system, there were only significant differences in telemedicine use by age group, with younger patients showing higher adoption (*P*<.001), and by racial or ethnic groups, with lower adoption by non-Hispanic Asian patients (*P*<.001). The safety net system showed significant differences in telemedicine use by racial and ethnic groups, with highest adoption by non-Hispanic Asian patients and lowest by non-Hispanic Black patients (*P*<.001), and by language preferences, with highest adoption by Chinese speakers (*P*<.001).

**Table 1:** Patient characteristics by health system among patients with any visits.

Patient characteristics			Combined health systems (N=10,201)							Academic system (n=3623)							Safety net system (n=6578)				
			In-person visits only		Telemedicine visit		*P* value		In-person visits only			Telemedicine visit		*P* value		In-person visits only			Telemedicine visit		*P* value
Patients, n (%)			1896 (19)		8305 (81)				973 (27)			2650 (73)				923 (14)			5655 (86)		
**Age group (years), n (%)**							<.001							<.001							.16
	18-34	26 (10)		223 (90)				10 (10)			90 (90)				16 (11)			133 (89)			
	35-49	228 (16)		1198 (84)				80 (19)			346 (81)				148 (15)			852 (85)			
	50-64	731 (18)		3375 (82)				285 (26)			797 (74)				446 (15)			2578 (85)			
	65-74	552 (20)		2192 (80)				320 (31)			712 (69)				232 (14)			1480 (86)			
	75+	359 (21)		1317 (79)				278 (28)			705 (72)				81 (12)			612 (88)			
**Race and ethnicity, n (%)**							.47							<.001							<.001
	Non-Hispanic White	306 (18)		1351 (82)				201 (22)			728 (78)				105 (14)			623 (86)			
	Non-Hispanic Asian	706 (19)		3026 (81)				459 (32)			971 (68)				247 (11)			2055 (89)			
	Non-Hispanic Black or African American	285 (19)		1192 (81)				119 (24)			371 (76)				166 (17)			821 (83)			
	Hispanic or Latine	469 (18)		2211 (83)				112 (26)			324 (74)				357 (16)			1887 (84)			
	Other or unknown	130 (20)		525 (80)				82 (24)			256 (76)				48 (15)			269 (85)			
**Language, n (%)**							<.001							.17							<.001
	English	1223 (21)		4541 (79)				768 (26)			2149 (74)				455 (16)			2392 (84)			
	Spanish	315 (16)		1630 (84)				28 (24)			91 (76)				287 (16)			1539 (84)			
	Chinese	176 (11)		1371 (89)				94 (32)			203 (68)				82 (7)			1168 (93)			
	Other or unknown	182 (19)		763 (81)				83 (29)			207 (71)				99 (15)			556 (85)			
**nSES^a^ quintiles, n (%)**							<.001							.26							.05
	1 (lowest)	537 (17)		2654 (83)				156 (24)			484 (76)				381 (15)			2170 (85)			
	2	421 (17)		2005 (83)				173 (25)			509 (75)				248 (14)			1496 (86)			
	3	324 (19)		1413 (81)				206 (29)			510 (71)				118 (12)			903 (88)			
	4	364 (21)		1396 (79)				251 (28)			632 (72)				113 (13)			764 (87)			
	5 (highest)	250 (23)		837 (77)				187 (27)			515 (73)				63 (16)			322 (84)			
Baseline hemoglobin A_1c_ (%), mean (SD)			7.5 (1.7)		7.6 (1.7)		.02		7.2 (1.3)			7.4 (1.6)		<.001		7.9 (2.1)			7.7 (1.8)		.02
**Blood pressure (mm Hg), n (%)**							.17							.91							.59
	≤120/80	402 (18)		1856 (82)				175 (26)			488 (74)				227 (14)			1368 (86)			
	≤140/90	992 (18)		4471 (82)				526 (27)			1451 (73)				466 (13)			3020 (87)			
	>140/90	413 (20)		1670 (80)				242 (27)			645 (73)				171 (14)			1025 (86)			
**Sex, n (%)**							<.001							.02							<.001
	Female	916 (17)		4,494 (83)				502 (25)			1480 (75)				414 (12)			3014 (88)			
	Male	980 (20)		3811 (80)				471 (29)			1170 (71)				509 (16)			2641 (84)			
**Insurance, n (%)**							<.001					.04							<.001		
	Private	448 (27)		1184 (73)				443 (28)			1124 (72)				5 (8)			60 (92)			
	Public	529 (17)		2512 (83)				102 (22)			354 (78)				427 (17)			2158 (83)			
	Medicare	721 (19)		3083 (81)				428 (27)			1172 (73)				293 (13)			1911 (87)			
	Uninsured	111 (15)		650 (85)				N/A^b^			N/A				111 (15)			650 (85)			
	Healthy Workers^c^	87 (9)		876 (91)				N/A			N/A				87 (9)			876 (91)			
**Patient portal enrollment, n (%)**							<.001							<.001							.007
	Activated	746 (22)		2655 (78)				685 (24)			2126 (76)				61 (10)			529 (90)			
**Charlson Comorbidity Index, n (%)**							.18							<.001							.06
	0-2	1531 (19)		6591 (81)				785 (29)			1879 (71)				746 (14)			4712 (86)			
	3+	365 (18)		1714 (82)				188 (20)			771 (80)				177 (16)			943 (84)			

^a^nSES: neighborhood socioeconomic status.

^b^N/A: not applicable.

^c^Healthy Workers is an insurance plan provided to temporary exempt employees of the City and County of San Francisco, including providers of in-home care.

### Adjusted Analyses

#### Combined Health System Data

**Figure 1:**
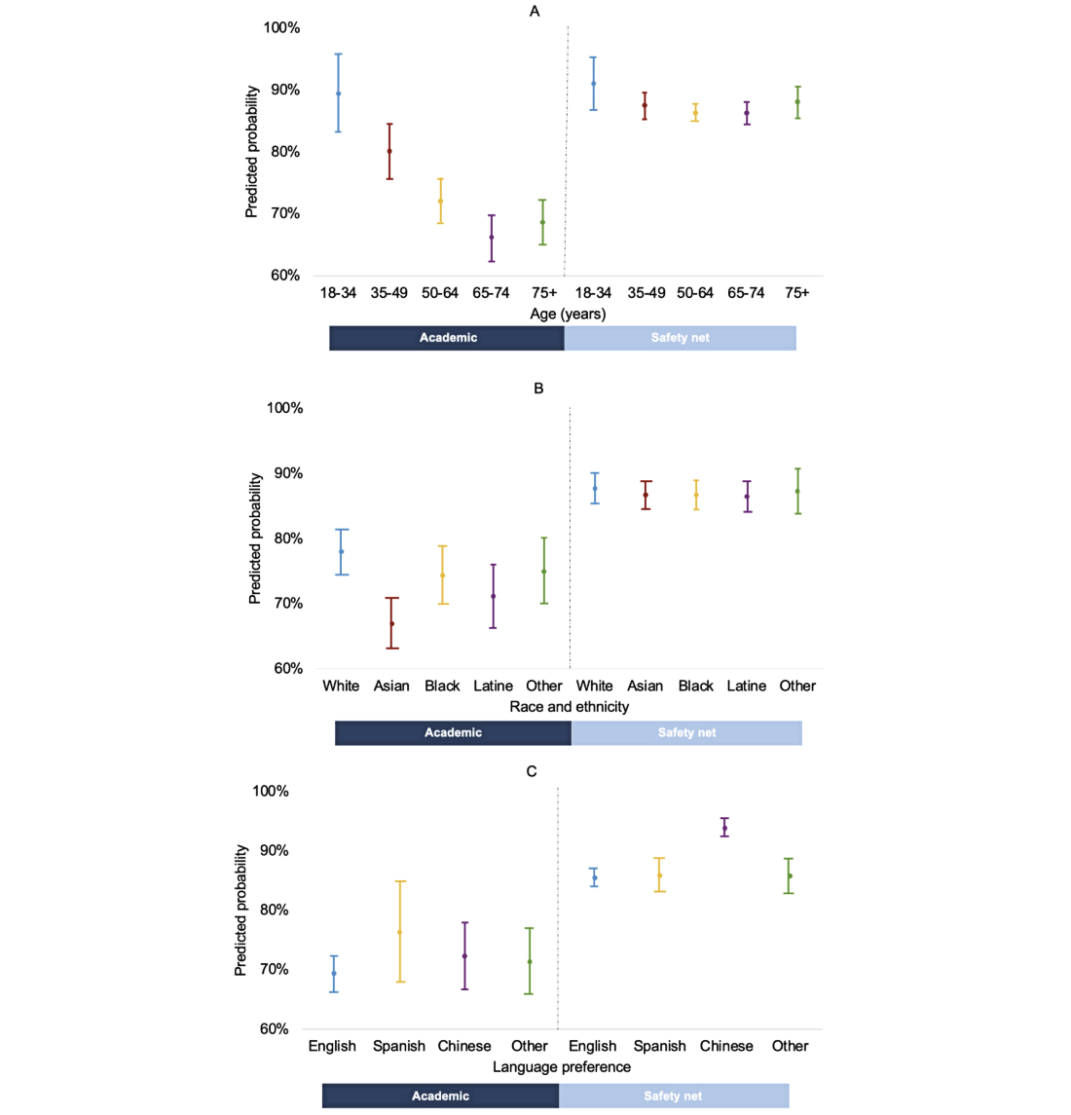
Predicted probabilities of telemedicine visits with 95% CIs for interactions between health system and (A) age, (B) race and ethnicity, and (C) language preference.

After adjusting for key predictors (age, race and ethnicity, language, and nSES) and covariates (baseline hemoglobin A_1c_, baseline BP, sex, insurance type, patient portal enrollment status, and CCI), safety net patients had higher odds of having a telemedicine visit compared with academic health system patients (aOR 2.94, 95% CI 2.48-3.48; Table 2). Patients aged 18-34 and 35-49 years also had higher odds of having a telemedicine visit than patients older than 75 years (aOR 2.55, 95% CI 1.63-3.97; aOR 1.39, 95% CI 1.12-1.73). Non-Hispanic Asian, non-Hispanic Black or African American, and Hispanic or Latine patients had lower odds of using telemedicine than non-Hispanic White patients (aOR 0.67, 95% CI 0.56-0.79; aOR 0.83, 95% CI 0.68-1.00; aOR 0.76, 95% CI 0.61-0.95, respectively), whereas patients with Chinese as their preferred language had higher odds of using telemedicine than those with an English-language preference (aOR 2.04, 95% CI 1.66-2.50). No significant differences were found among nSES. Results are displayed in Table 2. There was a significant interaction between health care system and each individual-level key predictor (*P*<.05; Multimedia Appendix 2). Health system significantly modified the effect of telemedicine use across age, racial and ethnic, and language preference groups, which is shown in Figure 1A-C, respectively. Estimated predicted probabilities for significant key predictors after applying the health system interaction term are provided in Multimedia Appendix 3. The interaction between health system and nSES was not statistically significant.

**Table 2:** Adjusted odds ratios of predictors of telemedicine use during the public health emergency.

Predictors		Combined health systems (N=10,201)			Academic system (N=3623)					Safety net system (N=6578)			
		aOR^a^	95% CI	aOR			95% CI		aOR			95% CI	
**Health system (reference: UCSF^b^)**													
	SFHN^c^	2.94^d^	2.48-3.48	N/A^e^			N/A		N/A			N/A	
**Age group (years; reference: 75+)**													
	18-34	2.55^d^	1.63-3.97	3.97^d^			1.99-7.93		1.42			0.78-2.60	
	35-49	1.39^f^	1.12-1.73	1.86^d^			1.36-2.56		0.98			0.70-1.37	
	50-64	1.09	0.92-1.30	1.15			0.91-1.45		0.87			0.65-1.16	
	65-74	0.95	0.81-1.12	0.91			0.74-1.11		0.84			0.64-1.11	
**Race and ethnicity (reference: White)**													
	Asian	0.67^d^	0.56-0.79	0.57^d^			0.46-0.70		0.88			0.67-1.17	
	Black or African American	0.83^g^	0.68-1.00	0.81			0.61-1.07		0.90			0.68-1.19	
	Hispanic or Latine	0.76^f^	0.61-0.95	0.67^g^			0.50-0.91		0.86			0.62-1.18	
	Other or unknown	0.89	0.70-1.13	0.86			0.63-1.16		0.94			0.64-1.38	
**Language (reference: English)**													
	Spanish	1.04	0.83-1.30	1.49			0.90-2.48		1.02			0.78-1.35	
	Chinese	2.04^d^	1.66-2.50	1.16			0.86-1.56		2.52^d^			1.85-3.42	
	Other or unknown	1.11	0.91-1.35	1.09			0.81-1.47		0.99			0.76-1.30	
**nSES^h^ quintiles (reference: 5)**													
	1	1.08	0.90-1.30	1.16			0.89-1.52		1.10			0.81-1.48	
	2	1.14	0.95-1.38	1.11			0.86-1.43		1.19			0.88-1.62	
	3	1.11	0.92-1.35	0.95			0.74-1.21		1.36			0.97-1.91	
	4	1.05	0.87-1.27	0.95			0.75-1.19		1.23			0.88-1.73	
Baseline hemoglobin A_1c_		1.03	0.99-1.07	1.11^d^			1.05-1.18		0.99			0.95-1.04	
**Baseline BP^i^ (mm Hg; reference: ≤120/80)**													
	≤140/90	1.08	0.95-1.23	1.04			0.85-1.28		1.10			0.93-1.31	
	>140/90	1.04	0.89-1.23	0.99			0.78-1.26		1.09			0.88-1.36	
**Sex (reference: male)**													
	Female	1.36^d^	1.22-1.51	1.36^d^			1.16-1.59		1.34^d^			1.15-1.55	
**Insurance type (reference: Medicare)**													
	Private	0.85^g^	0.72-1.00	0.82^g^			0.68-0.99		2.02			0.80-5.11	
	Public	0.84^g^	0.72-0.98	1.03			0.78-1.37		0.83			0.69-1.00	
	Uninsured	0.95	0.73-1.23	N/A			N/A		1.03			0.77-1.37	
	Healthy Workers^j^	1.35^g^	1.04-1.76	N/A			N/A		1.18			0.89-1.57	
**Patient portal status (reference: inactivated)**													
	Activated	1.71^d^	1.47-2.00			1.86^d^		1.55-2.23			1.46^f^		1.10-1.94
**Charlson Comorbidity Index (reference: 0-2)**													
	3+	1.33^d^	1.17-1.53			1.90^d^		1.57-2.30			0.93		0.77-1.12

^a^aOR: adjusted odds ratio.

^b^UCSF: University of California, San Francisco.

^c^SFHN: San Francisco Health Network.

^d^*P*<.001.

^e^N/A: not applicable.

^f^*P*<.01.

^g^*P*<.05.

^h^nSES: neighborhood socioeconomic status.

^i^BP: blood pressure.

^j^Healthy Workers is an insurance plan provided to temporary exempt employees of the City and County of San Francisco, including providers of in-home care.

#### Stratified Data by Health System

Given the significant interactions by health system, we analyzed each system independently. At the academic health system, participants in the younger age groups (aged 18-34 years and 35-49 years) had higher odds of having a telemedicine visit than those aged 75 years and older (aOR 3.97, 95% CI 1.99-7.93; and aOR 1.86, 95% CI 1.36-2.56, respectively; Table 2). In the academic health system, non-Hispanic Asian (aOR 0.57, 95% CI 0.46-0.70) and Hispanic or Latine (aOR 0.67, 95% CI 0.50-0.91) participants were less likely to have a telemedicine visit than non-Hispanic White participants. There were no significant differences in telemedicine utilization based on language preference or nSES in the academic health system.

On the other hand, in the safety net system, patients with a Chinese language preference had higher odds of having a telemedicine visit (aOR 2.52, 95% CI 1.85-3.42; Table 2). In the safety net system, there were no significant differences in telemedicine utilization based on age, race and ethnicity, or nSES. These findings were consistent with the interaction analysis (Figure 1).

## Discussion

### Principal Results

Our study explored differences in telemedicine uptake among a cohort of primary care patients with diabetes across 2 large, unaffiliated health systems in San Francisco, California, during the COVID-19 PHE. Our final adjusted results highlight that health system is a significant predictor of telemedicine use and that disparities in telemedicine utilization may arise from differing implementation strategies by health systems, such as prioritization of audio-only versus video visits. Potential inequities could arise if audio-only visits are not widely implemented or perceived as inferior, despite payor policies that are meant to increase their implementation. Health systems may need to tailor and evaluate telemedicine implementation strategies to address disparities in utilization and ensure equitable access to services, especially for populations at greatest risk for digital exclusion.

### Comparison With Prior Work

Our findings using the combined health system data are consistent with prior studies and indicated that groups traditionally vulnerable to digital exclusion (older and minoritized racial and ethnic groups) have lower odds of digital health uptake. These disparities in the context of telemedicine utilization have been well documented since the early stages of the PHE and have also been noted in the post-PHE era [6,11,18-22]. Novel to other studies, we found that these disparities differed across health systems with different telehealth implementation, even after adjustment for patient characteristics. When stratifying our models by health system, more disparities were found within the academic health system, where older, Asian, and Hispanic or Latine patients had lower odds of having a telemedicine visit than younger and non-Hispanic White patients, respectively; these disparities were not significant in the safety net system. In contrast, within the safety net system, Chinese-speaking patients had higher odds of having a telemedicine visit than other language preference groups.

### Offering Audio-Only Visits May Increase Telemedicine Accessibility

The safety net system evaluated in our study serves a diverse group of patients who are at an increased risk for digital exclusion [14]. Despite this challenge, its adoption of a telemedicine infrastructure that primarily uses audio-only visits may have mitigated potential disparities in telemedicine access. While in-person visits remain the gold standard for primary care, robust audio-only options can augment the accessibility of care to patients reliant on telemedicine [23]. Studies have suggested that patients are more satisfied with telehealth modalities overall compared with in-person visits in terms of access and care provided, with no preference between video and audio-only visits [24,25]. Audio-only visits may be more accessible than video visits due to increased ease of use and lower technological requirements such as high-speed web access, smartphones, and laptops. Excluding or limiting these audio-only options may disproportionately impair access for minoritized racial and ethnicity groups, older adult patients, and non–English-speaking patients.

Our findings may have implications for patients in rural areas. Like urban safety net systems, patient served by rural health care systems are often at risk for digital exclusion, including having limited broadband access, lower rates of device ownership, and lower rates of digital literacy, suggesting that audio-only telemedicine may be necessary to prevent exacerbating health access disparities in these regions [26].

### Considerations for Policy Makers and Health Care System Leaders to Ensure Telemedicine Equity

Our findings raise important considerations for future telemedicine policy, especially when considering the disparities observed within the academic health system. These disparities may partly be a consequence of the delayed and temporary approval for reimbursement of audio-only visits at a level similar to in-person visits by the Centers for Medicare & Medicaid Services [4]. The safety net system primarily served uninsured patients or those with public insurance (Medi-Cal) and Healthy Workers, where reimbursement rates were similar across audio-only and in-person visits. In contrast, the academic health system serves patients with private insurance or Medicare, both of which reimbursed video visits at lower rates than in-person or audio-only visits and thus had different incentives for telemedicine implementation. Inconsistent reimbursement across different telemedicine modalities can disproportionately impact populations at risk of digital exclusion and exacerbate existing health inequities. While video-enabled telemedicine care has been perceived in the United States to be more equivalent to in-person care and, therefore, reimbursed at a similar level for a longer period, audio-only telemedicine care has been perceived as providing lower quality and inadequate care, and therefore is reimbursed often at a much lower rate. This has resulted in limited motivation from many health care systems to provide audio-only encounters, which are more accessible to the populations that have experienced barriers to accessing traditional face-to-face health care and have limited digital literacy or access to digital tools [27].

Policy makers should assess the impact of reimbursement policies across different telemedicine modalities and payors to ensure equitable adoption and appropriate utilization among all patient groups, especially patients who may forgo care otherwise without accessible telemedicine modalities. While most payor are concerned that increased accessibility may lead to higher utilization and costs, payors should also recognize the value of this opportunity to improve health care access for historically excluded populations.

Policies for encouraging delivery of more accessible telemedicine services should be implemented in conjunction with policies that address the barriers to accessing video-based telemedicine care. This may include policies that increase affordability of web access and devices as well as targeted investments in digital literacy programs [28]. Community-based initiatives, such as mobile technology training program and device distribution efforts, may reduce barriers to telehealth adoption [29]. These programs can be scaled to address the needs of populations with low digital literacy or limited access to digital tools [29].

Health systems leaders need to evaluate both the technologic needs and the health care requirements of their populations when developing telemedicine strategies, ensuring accessibility and equity while adapting to evolving policies and regulations. Incorporating multiple telemedicine options into care models can improve accessibility for older adults, patients with language barriers, and those in areas with limited broadband connectivity.

Both policy makers and health care leaders must evaluate the impact of policies on health equity over time. Consistent, systematic data collection and reporting on telemedicine utilization and its impact on clinical outcomes by sociodemographic characteristic such as race and ethnicity, language preference, and insurance coverage can help identify persistent gaps and guide future policy. When policies and implementation methods are aligned with real-world patient needs, particularly for populations at high risk of digital exclusion, telemedicine can serve as a tool to effectively reduce health disparities.

### Limitations

Our sample included data from 2 urban health systems through March 2021, limiting generalizability to other systems and to later periods of the PHE. Future research evaluating the impact of telemedicine implementation in rural settings and examining trends in later post-PHE periods could further validate and broaden the applicability of these findings. However, more than 80% of the US population resides in urban areas, so our findings still have great relevance to many individuals [30]. In addition, differences in patient populations and institutional practices may be contributed to our findings. The safety net system may have had fewer disparities in telehealth use due to greater experience serving patients with limited digital access and stronger support for digital health literacy. However, our findings align with prior studies suggesting that telephone-based telehealth may improve accessibility and adoption among patient facing barriers to video-based services. We did not have access to the EHRs from outside systems, so we could not account for external encounters, although we limited our cohort to patients actively receiving primary care at UCSF or SFHN to mitigate this. Baseline measures of diabetes and BP control relied on single time points and may not perfectly reflect control through the study period. Incorporating longitudinal data in future analyses may help provide a more nuanced understanding of how disease control may be associated with telemedicine use.

### Conclusions

This retrospective study examined a cohort of 10,201 primary care patients with diabetes during the PHE and found that inequities in telemedicine utilization varied across health systems with differing telemedicine implementation. Notably, disparities related to patient age and racial and ethnic identity underscore potential barriers to health care access. Compared with the safety net system with widespread adoption of audio-only visits, patients in the academic health system were less likely to have any telemedicine visit. Furthermore, lower telemedicine utilization was observed among older patients and minoritized racial and ethnic groups in the academic health system—disparities that were not present in the safety net system, which serves a more diverse population. Ultimately, our findings suggest that rapid, widespread telemedicine implementation prioritizing video telemedicine may exacerbate disparities in telemedicine utilization. Providing both audio-only and video appointments may enhance accessibility to care for populations historically at risk for digital exclusion.

## Appendix

Multimedia Appendix 1 Patient characteristics by health system.

Multimedia Appendix 2 Predictors of telemedicine use during the public health emergency by patient characteristics and health system interaction (N=10,201).

Multimedia Appendix 3 Estimated predicted probabilities for significant key predictors of telemedicine use after applying the health system interaction term.

## Abbreviations

aOR: adjusted odds ratio
BP: blood pressure
CCI: Charlson Comorbidity Index
EHR: electronic health record
nSES: neighborhood socioeconomic status
PHE: public health emergency
SFHN: San Francisco Health Network
UCSF: University of California, San Francisco

## Data Availability

The data collected during this study are available from the corresponding author on reasonable request.
