# Supplementary material for: Differences in Telemedicine Use for Patients With Diabetes in an Academic Versus Safety Net Health System: Retrospective Cohort Study
Source: J Med Internet Res. 2025 Mar 24;27:e64635. doi: 10.2196/64635 (PMC11976178; doi:10.2196/64635)
Supplement: Multimedia Appendix 1 [file jmir_v27i1e64635_app1.docx]

|  | Academic | | Safety Net | | Combined | |
| --- | --- | --- | --- | --- | --- | --- |
|  | n | column % | n | column % | n | column % |
| Overall | 3,623 | 100% | 6,578 | 100% | 10,201 | 100% |
| Age | | | | | | |
| 18-34 | 100 | 3% | 149 | 2% | 249 | 2% |
| 35-49 | 426 | 12% | 1,000 | 15% | 1,426 | 14% |
| 50-64 | 1,082 | 30% | 3,024 | 46% | 4,106 | 40% |
| 65-74 | 1,032 | 28% | 1,712 | 26% | 2,744 | 27% |
| 75+ | 983 | 27% | 693 | 11% | 1,676 | 16% |
| Race/Ethnicity | | | | | | |
| NH White | 929 | 26% | 728 | 11% | 1,657 | 16% |
| NH Asian | 1,430 | 39% | 2,302 | 35% | 3,732 | 37% |
| NH Black or African American | 490 | 14% | 987 | 15% | 1,477 | 14% |
| Hispanic or Latine | 436 | 12% | 2,244 | 34% | 2,680 | 26% |
| Other/ Unknown | 338 | 9% | 317 | 5% | 655 | 6% |
| Language | | | | | | |
| English | 2,917 | 81% | 2,847 | 43% | 5,764 | 57% |
| Spanish | 119 | 3% | 1,826 | 28% | 1,945 | 19% |
| Chinese | 297 | 8% | 1,250 | 19% | 1,547 | 15% |
| Other/ Unknown | 290 | 8% | 655 | 10% | 945 | 9% |
| nSES Quintiles | | | | | | |
| 1 (lowest) | 640 | 18% | 2,551 | 39% | 3,191 | 31% |
| 2 | 682 | 19% | 1,744 | 27% | 2,426 | 24% |
| 3 | 716 | 20% | 1,021 | 16% | 1,737 | 17% |
| 4 | 883 | 24% | 877 | 13% | 1,760 | 17% |
| 5 (highest) | 702 | 19% | 385 | 6% | 1,087 | 11% |
| Baseline A1c (mean) | 7.3 |  | 7.8 |  | 7.6 |  |
| Blood Pressure | | | | | | |
| BP <=120/80 | 663 | 18% | 1,595 | 24% | 2,258 | 22% |
| BP <=140/90 | 1,977 | 55% | 3,486 | 53% | 5,463 | 54% |
| BP >140/90 | 887 | 24% | 1,196 | 18% | 2,083 | 20% |
| Sex | | | | | | |
| Female | 1,982 | 55% | 3,428 | 52% | 5,410 | 53% |
| Male | 1,641 | 45% | 3,150 | 48% | 4,791 | 47% |
| Insurance | | | | | | |
| Private | 1,567 | 43% | 65 | 1% | 1,632 | 16% |
| Public | 456 | 13% | 2,585 | 39% | 3,041 | 30% |
| Medicare | 1,600 | 44% | 2,204 | 34% | 3,804 | 37% |
| Uninsured | N/A | N/A | 761 | 12% | 761 | 7% |
| Healthy Workers^a^ | N/A | N/A | 963 | 15% | 963 | 9% |
| Patient Portal Enrollment | | | | | | |
| Activated | 2,811 | 78% | 590 | 9% | 3,401 | 33% |
| Charlson Comorbidity Index | | | | | | |
| 0-2 | 2,664 | 74% | 5,458 | 83% | 8,122 | 80% |
| 3+ | 959 | 26% | 1,120 | 17% | 2,079 | 20% |

^a^Healthy Workers is an insurance plan provided to temporary exempt employees of the City and County of San Francisco, including providers of in-home care.
