# Supplementary material for: Differences in Telemedicine Use for Patients With Diabetes in an Academic Versus Safety Net Health System: Retrospective Cohort Study
Source: J Med Internet Res. 2025 Mar 24;27:e64635. doi: 10.2196/64635 (PMC11976178; doi:10.2196/64635)
Supplement: Multimedia Appendix 2 [file jmir_v27i1e64635_app2.docx]

|  | aOR | 95% CI |
| --- | --- | --- |
| Health System |  |  |
| SFHN | 2.34*** | (1.45, 3.77) |
| Age Groups (ref. 75+) |  |  |
| 18-34 | 3.98*** | (2.01, 7.88) |
| 35-49 | 1.87*** | (1.38, 2.55) |
| 50-64 | 1.18 | (0.95, 1.47) |
| 65-74 | 0.89 | (0.73, 1.09) |
| *Interaction Term* |  |  |
| 18-34*SFHN | 0.35* | (0.14, 0.85) |
| 35-49*SFHN | 0.51*** | (0.33, 0.78) |
| 50-64*SFHN | 0.72 | (0.51, 1.01) |
| 65-74*SFHN | 0.95 | (0.68, 1.33) |
| Race/ethnicity (ref. White) |  |  |
| Asian | 0.56*** | (0.45, 0.69) |
| Black/African American | 0.81 | (0.62, 1.08) |
| Hispanic/Latine | 0.69* | (0.51, 0.93) |
| Other/Unknown | 0.84 | (0.62, 1.14) |
| *Interaction Term* |  |  |
| Asian*SFHN | 1.63** | (1.15, 2.30) |
| Black/African American*SFHN | 1.12 | (0.76, 1.66) |
| Hispanic/Latino*SFHN | 1.29 | (0.83, 2.00) |
| Other/Unknown*SFHN | 1.14 | (0.70, 1.86) |
| Language (ref. English) |  |  |
| Spanish | 1.43 | (0.87, 2.37 |
| Chinese | 1.15 | (0.86, 1.55) |
| Other/Unknown | 1.10 | (0.82, 1.48) |
| *Interaction Term* |  |  |
| Spanish*SFHN | 0.72 | (0.41, 1.27) |
| Chinese*SFHN | 2.26*** | (1.48, 3.45) |
| Other/Unknown*SFHN | 0.93 | (0.63, 1.38) |
| nSES Quintiles (ref. 5) |  |  |
| 1 | 1.19 | (0.91, 1.55) |
| 2 | 1.14 | (0.89, 1.46) |
| 3 | 0.96 | (0.76, 1.23) |
| 4 | 0.97 | (0.77, 1.22) |
| *Interaction Term* |  |  |
| 1*SFHN | 0.93 | (0.62, 1.39) |
| 2*SFHN | 1.06 | (0.71, 1.57) |
| 3*SFHN | 1.44 | (0.95, 2.18) |
| 4*SFHN | 1.29 | (0.86, 1.95) |
| Baseline A1c | 1.03 | (0.99, 1.07) |
| Baseline BP (ref. <=120/80) |  |  |
| BP <=140/90 | 1.08 | (0.94, 1.23) |
| BP >140/90 | 1.05 | (0.89, 1.23) |
| Sex (ref. Male) |  |  |
| Female | 1.34*** | (1.21, 1.49) |
| Insurance Type (ref. Medicare) |  |  |
| Private | 0.80** | (0.68, 0.95) |
| Public | 0.89 | (0.76, 1.04) |
| Uninsured | 1.10 | (0.84, 1.45) |
| Healthy Workers^1^ | 1.28 | (0.98, 1.68) |
| Patient Portal Status (ref. inactivated) |  |  |
| Activated | 1.70*** | (1.46, 1.99) |
| Charlson Comorbidity Index (CCI, ref. 1-2) |  |  |
| 3+ | 1.35*** | (1.18, 1.54) |
| Constant | 1.41 | (0.94, 2.09) |

^1^Healthy Workers is a San Francisco-provided healthcare insurance for home caregivers.

*p<0.05, **p<0.01, ***p<0.001
