# Supplementary material for: Differences in Telemedicine Use for Patients With Diabetes in an Academic Versus Safety Net Health System: Retrospective Cohort Study
Source: J Med Internet Res. 2025 Mar 24;27:e64635. doi: 10.2196/64635 (PMC11976178; doi:10.2196/64635)
Supplement: Multimedia Appendix 3 [file jmir_v27i1e64635_app3.docx]

| Health System |  | Predicted Probability | 95% CI |
| --- | --- | --- | --- |
|  | Age |  |  |
| UCSF | 18-34 | 0.89 | (0.83, 0.96) |
|  | 35-49 | 0.80 | (0.76, 0.85) |
|  | 50-64 | 0.72 | (0.68, 0.76) |
|  | 65-74 | 0.66 | (0.62, 0.70) |
|  | 75+ | 0.69 | (0.65, 0.72) |
| SFHN | 18-34 | 0.91 | (0.87, 0.95) |
|  | 35-49 | 0.88 | (0.85, 0.90) |
|  | 50-64 | 0.86 | (0.85, 0.88) |
|  | 65-74 | 0.86 | (0.85, 0.88) |
|  | 75+ | 0.88 | (0.86, 0.91) |
|  | Race/Ethnicity |  |  |
| UCSF | White | 0.78 | (0.74, 0.82) |
|  | Asian | 0.67 | (0.63, 0.71) |
|  | Black/African American | 0.74 | (0.70, 0.79) |
|  | Hispanic or Latino | 0.71 | (0.66, 0.76) |
|  | Other/Unknown | 0.75 | (0.70, 0.80) |
| SFHN | White | 0.88 | (0.85, 0.90) |
|  | Asian | 0.87 | (0.85, 0.89) |
|  | Black/African American | 0.87 | (0.85, 0.89) |
|  | Hispanic | 0.86 | (0.84, 0.89) |
|  | Other/Unknown | 0.87 | (0.84, 0.91) |
|  | Language Preference |  |  |
| UCSF | English | 0.69 | (0.67, 0.72) |
|  | Spanish | 0.76 | (0.68, 0.85) |
|  | Chinese | 0.72 | (0.67, 0.78) |
|  | Other/Unknown | 0.71 | (0.66, 0.77) |
| SFHN | English | 0.85 | (0.84, 0.87) |
|  | Spanish | 0.86 | (0.83, 0.89) |
|  | Chinese | 0.94 | (0.92, 0.95) |
|  | Other/Unknown | 0.86 | (0.83, 0.89) |
